# Identifying Gifted Children: Congruence among Different IQ Measures

**DOI:** 10.3389/fpsyg.2017.01239

**Published:** 2017-07-20

**Authors:** Estrella Fernández, Trinidad García, Olga Arias-Gundín, Almudena Vázquez, Celestino Rodríguez

**Affiliations:** ^1^Faculty of Psychology, Oviedo University Oviedo, Spain; ^2^Department of Psychology, Sociology and Philosophy, Faculty of Education, León University León, Spain; ^3^Asunción León-Primary and Secondary School León, Spain

**Keywords:** intellectual ability, creativity, primary school, high ability, assessment methods

## Abstract

This study has two main aims: (1) analysing the relationship between intellectual capacities and levels of creativity in a sample of Spanish students from the third and sixth grades; and (2) examining the discrimination capacities and degree of congruence among different tests of intellectual ability that are commonly used to identify high-ability students. The study sample comprised 236 primary school students. Participants completed different tests of intellectual ability, which were based on both fluid and crystallized intelligence, as well as creativity. Results indicated that it is advisable to use varying tests in the assessment process, and a complementary measure (i.e., creativity) in order to create a multi-criteria means of detection that can more efficiently distinguish this population of students.

## Introduction

Identifying students with higher abilities has become a subject of great interest for researchers, education administrators, teachers and families alike. However, it is also a controversial issue because there is still no agreement on which variables must be taken into account to determine whether a student has higher abilities, or how these variables should be measured in these cases.

The different conceptualizations of higher intellectual abilities, either from educational, socio-political or psychometric perspectives, have traditionally tried to identify those children who are exceptional (Pfeiffer, 2015). One of the models that has received more attention is the Three-Ring Conception of Giftedness by Renzulli (1978). This model has helped establish some of the general criteria being used to classify students with higher abilities today. This author defined high intellectual ability as a consistent interaction between three basic human traits that characterize high-ability people: (a) above-average general intelligence; (b) creativity (defined as “that cluster of traits that encompasses curiosity, originality, ingenuity, and a willingness to challenge convention and tradition”; and (c) task commitment (which “represents a non-intellective cluster of traits found consistently in creative and productive individuals, including perseverance, determination, will power or positive energy”) (Renzulli, 2012). This model has been used as a reference in Spanish schools to determine which students are gifted and which students are not gifted. In which the creativity acquiring, at a practical level, great protagonism, above-average commitment. Moreover, some studies show that gifted learners are more creative than average learners, for example, when evaluating divergent thinking or amount of original ideas (Ferrando et al., 2008; Jauk et al., 2013).

However, this is not the only model to be considered. Other authors such as Jeltova and Grigorenko (2005), Calero et al. (2007), and Pfeiffer, (2012) consider high-ability children as those who demonstrate a higher likelihood of attaining significant achievements in culturally valued domains. These authors take into account a student’s intellectual abilities, while also emphasizing the relevance of certain personality traits and the role of stimulating social environments that can effectively favor an individual’s learning in specific fields. However, regardless of the theoretical model, there is agreement today that higher intellectual ability is a multi-dimensional construct, and that more human and material resources are needed to identify this often-latent potential in order to provide appropriate educational support to such students (Tourón et al., 1998; Pfeiffer, 2015). It is therefore fundamental that schools and professionals are provided with the right tools to identify high-ability students as early as possible (Reis and Renzulli, 2010).

Traditionally, intellectual ability was the central variable used to discriminate high-ability individuals from the average population. Nowadays, however, various authors agree that intellectual quotient (IQ) cannot be used as a single variable in the conceptualization of high abilities (Calero and García-Martín, 2011; Pfeiffer, 2015). For example, as discussed by Wellisch and Brown (2012) in their study, some authors suggest that the most reliable information would be based on the perception of teachers and families. Nevertheless, IQ remains an important factor to be assessed and, when used in conjunction with other variables, it can provide essential information concerning the identification of students with exceptional abilities (Sternberg, 2010; Renzulli and Gaesser, 2015). Moreover, many educational policies establish that, in order to implement effective identification and intervention processes, a non-negotiable criterion is to evaluate the student’s intellectual capacity by means of standardized tests (Wet and Gubbins, 2011). Although other criteria may be used, there are currently authors who consider that these criteria cannot equal the objectivity and reliability of IQ measurements and tasks, especially for students with learning difficulties (Lovett and Lewandowski, 2006). This broader approach to assessment is important, since the responsibility of detecting high-ability students often falls to schools, which commonly only pay attention to the more traditional signals related to high-ability, such as high levels of academic achievement. Evaluation and intervention recommendations come from teachers in most cases (Renzulli and Gaesser, 2015); however, most teachers do not have a vast knowledge in the identification of high-ability students. This may lead to mistakes during the assessment process (Tourón et al., 2006; Reis and Renzulli, 2010) and under-identification of some students, especially those from lower socio-economic backgrounds (Moon and Brighton, 2008; Baker, 2011; Freeman, 2011; Wellisch and Brown, 2012), and/or those who have socio-emotional problems and may appear to have low levels of competence in basic learning processes (emulating students with learning difficulties) (Silverman, 2009; Wellisch and Brown, 2012).

Therefore, although the exclusive use of standardized tests to assess intellectual ability has its detractors (Pfeiffer, 2012) and these tests are not the only measures available nowadays, the fact remains that standardized tests have been accepted as reliable measures of identifying students with higher abilities to date (Lovett and Lewandowski, 2006; Lovett and Sparks, 2011; Erwin and Worrell, 2012) and as Carman (2013) suggests “no matter how often researchers suggest that an IQ score is not the only way of determining giftedness, it is still the most common method of identifying gifted participants for research, either alone or in combination with other criteria.” At a practical level, in Spain the information obtained from standardized tests is the first criterion used to determine if a student may have higher abilities, and is essential for continuation of the evaluation process. This measure is used as a baseline analysis of the students’ capacities and offers a starting point for the detection of higher intellectual abilities (Renzulli, 2012; Wellisch and Brown, 2012).

Accepting this condition as necessary, a new problem arises concerning which standardized tests to choose and the degree of congruence required between different measures. This difficulty is associated, in part, with the definition of intelligence itself and with the variables that are considered relevant to measure this construct (e.g., abstract reasoning, vocabulary, numerical knowledge). Standardized tests designed to evaluate the IQ are based on different conceptualizations of intelligence and this is an important aspect to consider when deciding which measure should be used. Some authors recommend the use of non-verbal tests to avoid cultural and linguistic biases (Naglieri and Ford, 2003) such as the *Factor “g”* test (Cattell and Cattell, 1994) or “Matrices” (Sánchez-Sánchez et al., 2015), both of which are considered good estimators of fluid intelligence and general intellectual ability (or “g” factor). Other authors, in order to provide a more contextual perspective to the conceptualization of the intelligence, give greater weight to the evaluation of psychological variables relevant to the execution of school tasks, thus estimating intellectual ability by focusing on school competences rather than on purely intellectual capacities (Thurstone and Thurstone, 2005). Finally, some authors state that appropriate testing should take the form of batteries of tests that also collect information on a wide range of variables that, in the last decades, have demonstrated they are good indicators of intelligence, such as students’ verbal competence, together with components such as working memory, processing speed, comprehension, analytical capacity, and so forth (Sternberg, 2010; Pierson et al., 2012).

At this point it is worth noting the current interest in the research community in hierarchical models of intelligence and their tests, and specifically in the Cattell–Horn–Carroll Theory of Cognitive Abilities (CHC) (McGrew, 2005). This theory establishes three strata in the conceptualization of intelligence: stratum III – general or global intelligence; stratum II (broad) – 10 general intelligence abilities which are the main focus of interest in the assessment of intellectual ability and are fluid and crystallized intelligence, short-term or immediate memory, long-term memory storage and retrieval, processing speed, quantitative reasoning, reacting or decision making speed, visual processing, auditory processing, reading ability, and writing ability; and stratum I (narrow) – made up of more specific components such as inductive processes, vocabulary, visual memory, spatial relations, and general sequential reasoning, and which would conform to the general cognitive factors of stratum II.

Although this theory is gradually having an impact on the evaluation and identification of higher ability students at the international level (Pfeiffer, 2015), and new assessment tools are being designed or adapted based on this model (e.g., WISC-V; Wechsler, 2014), at a practical level, at least in Spain, it has not yet become established as a specific assessment protocol adjusted to this perspective. Therefore, both the detection model and the tests used ultimately depend on the experience and knowledge of the professionals in charge of the evaluation, and the assessment measures available in each case.

The present study had two objectives. First, following Renzulli’s (1978) model, it aimed to describe intellectual capacities and creativity levels of a sample of primary school students from northern Spain, with the aim of detecting and analysing potential cases of high ability where IQ is 130 or above – or two typical deviations above the average. Students from grades 3 and 6 were chosen as representative of this stage, and two variables of measures, intellectual capacity and creativity, were measured. Second, taking into account that depending on the tests used the students identified as gifted children may be different, this study aimed to establish the congruence and efficacies of different types of intellectual ability measures in order to determine if they concur, with respect to distinguishing students with higher abilities from average students. In schools it is common to use only a test of intellectual capacity in the processes of identification. Therefore, it is necessary to determine if these results in incorrect identification, either by over- or under-identification, due to inconsistencies between different type tests results.

In this analysis, although they are important variables in Renzulli’s (1978) model, task involvement and academic performance are not included as discriminating criteria because previous literature suggests that many students with high ability fail in the academic environment due to related factors, such as lack of motivation, and poor recognition by teachers of their real educational needs, both of which can also arise due to “teacher-bias” (Reis and Renzulli, 2004, 2009).

## Materials and Methods

### Participants

A sample of 236 primary school students from northern Spain took part in this study. The students were recruited from the third grade (*n* = 117; 49.6%) and the sixth grade (*n* = 119; 50.4%). Their ages ranged from 8 to 13 years (*M* = 9.96; *SD* = 1.65). The ratio of males to females in the total sample was not ideal (χ^2^ = 4.90; *p* = 0.027). There were no statistically significant differences in the percentage of students in the different grades (*p* = 0.90). The ages of the third grade students ranged from 9 to 10 years (*M* = 8.38; *SD* = 0.51), with 63 (53.8%) of the sample being female, and 54 (46.2%) being male. There were no statistically significant differences regarding gender distribution (*p* = 0.405). In the case of the sixth grade students, their ages ranged from 11 to 13 years (*M* = 11.50; *SD* = 0.55), with 47 (39.5%) being female and 72 (60.5%) being male. There were statistically significant differences between the proportion of boys and girls in this group (χ^2^ = 5.25; *p* = 0.022).

### Measures

The following instruments were administered:

#### Intellectual Abilities

Three measures traditionally used in the assessment of intelligence were used. The *Test of Educational Aptitudes* (TEA-1) is a test of academic competences based on a selection of the most relevant factors from the “Primary Mental Abilities” by Thurstone (1938). The *Battery of Differential and General Skills* (Badyg) is consistent with the Cattell–Horn–Carroll theory (CHC) as the test is based on a hierarchical model of intelligence with three different levels. Lastly, the *Factor “g”* test is a non-verbal test which provides a measure of fluid intelligence (Gf) and general intellectual ability, or g factor. Due to the age of the students, two different versions of the Badyg were used. Specifically, students in grade 3 completed the Badyg-2, while students in grade 6 completed the Badyg-3. A more detailed description of these tests follows.

Test of Educational Aptitudes (adapted to Spanish by Department I+D of TEA Editions, S.A.) (Thurstone and Thurstone, 2005) test provides an estimation of general intelligence and its factors. It consists of five parts that measure three different components or abilities (i.e., factors): verbal (different words and vocabulary), numerical (calculation), and reasoning (drawing and series). It also offers the possibility to measure verbal and non-verbal abilities separately. It is available in three different versions for different age groups. The TEA-1 version was used in the present study and was administered according to the age range of the sample. Reliability coefficients by mean of Cronbach’s alpha ranged between 0.61 and 0.95 for the different subtests, with an alpha of 0.89 for the full scale. The manual reports adequate internal validity, although correlations between different variables are mostly low to moderate. High correlations are only reported between verbal reasoning and academic aptitude (*r* = 0.89), and between academic aptitude and numerical reasoning (*r* = 0.85).

Battery of Differential and General Skills (Badyg) (Yuste et al., 2005) provides an estimation of IQ and presents different versions for different age groups. Students in sixth grade completed the Badyg-E3, which consists of six subtests: (1) analog relations (verbal intelligence), (2) numerical series (inductive reasoning), (3) matrices (fluid intelligence), (4) sentence completion (inductive reasoning), (5) numerical problems (verbal intelligence), and (6) figure matching (visual processing). An overall full-scale IQ index score is also provided. Students in third grade completed the Badyg-E2. It is made up of the same subtests as the Badyg-E3 but varies in difficulty level and application time. Cronbach’s alpha was from 0.77 to 0.84 for the different subtests, and 0.95 for the full scale. The Cronbach’s alpha obtained in the present study, for the full scale, was 0.72.

While there are more powerful assessment tools to evaluate this component and with better psychometric properties, this instrument was chosen for the following reasons: (a) it can be used to predict academic performance in a reliable way; (b) it has been used in previous studies which demonstrated a relationship between intellectual ability and academic performance; and (c) factorial analysis showed high correlations between the different sub-scales that compose the Badyg battery. Criterion validity was moderate to high (Pearson’s *r* from 0.39 to 0.58). This scale also shows a well-adjusted factorial structure making it possible to carry out additional broad-scoped comparisons (e.g., Sabiston et al., 2013).

The *Factor “g”* test (Cattell and Cattell, 1994 – adapted to Spanish by Associated Specialized Technicians) evaluates intelligence conceived as a general mental ability. It uses non-verbal tasks to eliminate the influence of those abilities that have been acquired through education, such as vocabulary or numerical knowledge. This test has three versions, each with different difficulty levels. The selection of the level depends upon the age of the participant. Level 2 (suitable for children from 8 to 14 years) was used in the present study. It includes four subtests: series, classification, conditions, and matrices. Individual scores are combined to obtain a global IQ score. The participant is asked to establish logical relationships between abstract figures and forms.

Cronbach’s alpha ranged between 0.76 and 0.85 for the different subtests (alpha = 0.86 for the full scale), with a complementary index adequate stability of 2.59 (typical measurement errors). Criterion validity was high, finding statistically significant correlations between the different sub-scales and the Test of Educational Aptitudes-TEA 1 and 2 (Pearson’s *r* from 0.53 to 0.81; *p* < 0.001).

#### Creativity

The *Creative Intelligence Test* (CREA) (Corbalán et al., 2003) presents participants with an image (commonly representing a social scene) and they have a limited time frame to formulate all the questions that the situation evokes in them. Version C, which is aimed at children, was used. In addition to providing a global measure of creativity, it offers the possibility to analyze the results qualitatively. Three levels of creativity can be established based on percentages (low = below the 25th percentile; medium = 26th–74th percentiles; and high = 75th percentile and above).

### Procedure

Students were recruited from different schools in Northern Spain. Once the schools were selected, principals and head teachers of the participating schools were contacted. They were informed about the aims of the study, its voluntary nature and anonymity, and the ethical treatment of the data recorded. The study was conducted in accordance with The Code of Ethics of the World Medical Association (Declaration of Helsinki), which reflects the ethical principles for research involving humans (Williams, 2008). Informed consent from families was also obtained. Researchers who were trained in psychology administered the above tests, all of which were conducted using counter-balanced methodology over the course of the testing, in three different testing sessions. Students with severe learning difficulties or special educational needs were excluded from the analyses.

### Data Analysis

A descriptive design was used. Due to the objectives of this study, statistical analyses were performed in different steps. First, the sample was described in terms of age, gender, IQ (based on the three measures of intelligence previously described), and creativity. This analysis was conducted separately for students in grade 3 and 6, as different versions of the Badyg were used. The normality of the dependent variables (i.e., global scores in the CREA, Badyg, TEA-1, and *Factor “g”* test) was analyzed, paying special attention to skewness and kurtosis values. Following Finney and Di Stefano’s (2006) criterion, the adequacy of these values was demonstrated (**Table 1**). Secondly, to estimate the correspondence between the different measures of intellectual ability, Pearson correlation between global IQ scores were conducted.

**Table 1:** Descriptive statistics for the sample (third and sixth grade students).

	Third grade students (*N* = 117)			Sixth grade students (*N* = 119)		
		*M*	*SD*		*M*	*SD*
CREA-Q	Low *n* = 29 (24.8%)			Low *n* = 5 (4.2%)		
	Medium *n* = 69 (59%)			Medium *n* = 66 (55.5%)		
	High *n* = 19 (16.2%)			High *n* = 48 (40.3%)		
CREA-RS		7.94	3.81		10.85	3.60
Factor *g* test		109.28	15.90		94.27	20.87
BADYG		102.03	16.96		99.94	16.71
TEA-1		101.79	12.94		104.35	16.17

*CREA-Q, CREA qualitative: low, medium, and high creativity; CREA-RS, CREA raw score; n, number of students by level; M, Mean; SD, Standard Deviation*.

Additionally, student’s *t*-test was also performed to analyze within-subject differences in IQ estimated with the different tests. To analyze the discriminatory capacity of each test in the detection of students with high abilities, the absolute frequency of students with an IQ of 130 or higher (as determined by the different tests) was then calculated. The congruence among the three intelligence tests was estimated by recording the number of students who were found to have an IQ of 130 or above in all the tests. Congruence between pairs of tests in the detection of high-ability students was also established. Although considering an IQ of 130 or above – or two typical deviations above the average – seems to be an arbitrary criterion, in both research and educational practice this criterion is still used, in most cases, as a cut-off point to determine which students have higher intellectual abilities (Moon and Brighton, 2008; Carman, 2013; Guignard et al., 2016; Peyre et al., 2016).

## Results

### Intellectual IQ Results of the Students and Correspondence between Measures

**Table 1** shows descriptive statistics for the sample, while **Table 2** presents correlations between IQ scores measured using the different tests of intellectual ability described. Analyses for students in grade 3 and 6 are presented separately.

**Table 2:** Bivariate correlations between IQ scores in the different tests (third and sixth grade students).

	Third grade students (*N* = 117)				Sixth grade students (*N* = 119)		
	Factor *g* test	Badyg-2	TEA-1		Factor *g* test	Badyg-3	TEA-1
Factor *g* test		0.605^∗^	0.373^∗^	Factor *g* test		0.159	0.031
Badyg-2			0.502^∗^	Badyg-3			0.746^∗^
TEA-1				TEA-1			

*^∗^p < 0.001.*

#### Third Grade Students

As **Table 1** shows, 59% of the students in grade 3 had a medium level of creativity, while only 19% reached high levels of creativity. However, the mean in this variable suggests low levels of creativity in general (values in this variable can range from 0 to 25).

Results from the intelligence tests administered placed the intellectual ability of the group around the average, regardless of the test used. Scores were slightly higher in the case of the *Factor “g”* test (i.e., fluid intelligence). Standard deviations were high, suggesting the presence of large inter-subject variability. IQ values ranged from 68 to 149 points in the case of the *Factor “g”* test, between 65 and 135 in the TEA-1, and between 64 and 139 in the Badyg-2. The correlations between the various measures of intellectual ability were positive and statistically significant between all pairs of tests (see **Table 2**). Statistically significant differences between IQ scores estimated with *Factor “g”* test and Badyg-2 (*t* = 5.369; *p* < 0.001), and between *Factor “g”* test and TEA-1 (*t* = 4.964; *p* < 0.001) were found, but not between the Badyg-2 and TEA-1 (*p* = 0.866). Thus, statistically significant differences were found when the crystallized and fluid intelligence measures were compared, with students’ IQ scores being higher when using the latter measure.

#### Sixth Grade Students

Results show that students in this group obtained higher scores in CREA than the younger students. However, the scores varied from a minimum of 4 to a maximum of 20 in this variable. Again, the proportion of students with medium creativity was greater than the proportion of students with low and high creativity. However, the percentage of students with high levels of creativity was greater than in the third grade students group (see **Table 1**).

Regarding the variable IQ, sixth grade students showed average levels of intelligence, although a large within-subject variability was observed. IQ scores ranged from 30 to 139 points when the *Factor “g”* test was used, from 55 to 136 in the case of the Badyg-3, and from 65 to 135 when the TEA-1 was administered. Correlations between the different measures were positive, but only statistically significant when using the Badyg-3 and TEA-1 (see **Table 2**). At a within-subject level, statistically significant differences in IQ scores were observed when the *Factor “g”* test and Badyg-3 were compared (*t* = -2.529; *p* = 0.013), as well as between the *Factor “g”* test and TEA-1 (*t* = -4.237; *p* < 0.001), and between the Badyg-3 and TEA-1 (*t* = -4.092; *p* < 0.001). Students in grade 6 obtained better results in the TEA-1 than in the other tests.

### Discriminatory Values of the Measures in the Detection of Students with High Abilities, and the Intellectual Measures of the Students Detected

To detect students that could be considered high-ability and determine the congruence between the tests, a selection of cases in which a student scored 130 or above in the different IQ tests was made. Results are presented according to school grade (**Tables 3**, **4**).

**Table 3:** Descriptive statistic of participants with an IQ equal or above 130 in the different tests (third grade students).

Age	Gender	CREA-Q			CREA-RS	Factor *g* test	Badyg-2	TEA 1
*M* (*SD*)	M/F	Low	Medium	High	*M (SD)*	*M* (*SD*)	*M* (*SD*)	*M* (*SD*)
*Convergence between Factor g test and Badyg-2 (n = 2)*								
8.5 (0.707)	1M/1F	–	*n* = 1 (50%)	*n* = 1 (50%)	10.501 (2.242)	139 (9.898)	137.5 (2.123)	125.5 (6.363)
*IQ above or equal to 130 in Factor g test (n* = *13)*								
8.38 (0.506)	7M/6F	*n* = 2 (15.4%)	*n* = 10 (76.9%)	*n* = 1 (7.7%)	8.461 (2.781)	136.232 (6.300)	116.846 (14.512)	107.676 (14.332)
*IQ above or equal to 130 in Badyg-2 (n* = *6)*								
8.667 (0.516)	3M/3F	*n* = 1 (16.7%)	*n* = 4 (66.6%)	*n* = 1 (16.7%)	8.667 (3.265)	128 (1.714)	132 (3.982)	118.16 (7.935)
*IQ above or equal to 130 in TEA 1 (n* = *1)*								
9	1F	–	*n* = 1 (100%)	–	8	122	127	135

*M/F, male/female; CREA-Q, CREA qualitative: low, medium, and high creativity; CREA-RS, CREA raw score; M, Mean; SD, Standard Deviation.*

**Table 4:** Descriptive statistic of participants with an IQ equal or above 130 in the different tests (sixth grade students).

Age	Gender	CREA-Q			CREA-RS	Factor *g* test	Badyg-3	TEA-1
*M* (*SD*)	M/F	Low	Medium	High	*M* (*SD*)	*M* (*SD*)	*M* (*SD*)	*M* (*SD*)
*Convergence between Factor g test and TEA 1 (n = 1)*								
12	1M	–	–	*n* = 1 (100%)	19	139	127	135
*IQ above or equal to 130 in Factor g test (n* = *4)*								
11.5 (0.577)	2M/2F	–	*n* = 1 (25%)	*n* = 3 (75%)	14.750 (3.862)	132.750 (4.193)	117.250 (7.500)	110.250 (17.967)
*IQ above or equal to 130 in Badyg-3 (n* = *1)*								
12	1F	–	*n* = 1 (100%)	–	9	122	136	119
*IQ above or equal to 130 in TEA 1 (n* = *6)*								
11.5 (0.547)	4M/2F	–	*n* = 3 (50%)	*n* = 3 (50%)	12.833 (3.656)	101 (22.172)	116.500 (9.995)	135

*M/F, male/female; CREA-C, CREA qualitative: low, medium, and high creativity; CREA-RS, CREA raw score; M, Mean; SD, Standard Deviation.*

#### Third Grade Students

None of the students in this group obtained an IQ score of 130 or above in all three of the tests. However, scores from the *Factor “g”* test and Badyg-2 converged in two cases. With respect to the other possible paired-comparisons of the tests, there were no instances of converging results (**Table 3**). These students showed a medium-to-high percentile in the creativity test and a mean IQ of 139 points in the *Factor “g”* test, with values ranging from 132 to 146 points. They also exhibited a mean of 137.5 points in the Badyg-2, with values between 136 and 139. Regarding IQ assessed by the TEA-1, values were close to 130, ranging from 119 to 128 points.

For students who had an IQ of 130 or greater in only one of the tests, it can be observed that the *Factor “g”* test identified the highest number of students who met this criterion (13 students), while the TEA-1 was the most restrictive test with only one student identified. The Badyg-2, however, detected six students who met the above-mentioned criterion. It should be noted that, in the majority of cases, students identified as having high-abilities showed a medium level of creativity. Finally, IQ scores ranged between 130 and 149 points when measured by the *Factor “g”* test, and between 130 and 139 in the case of the Badyg-2. A unique value of 135 was found in the TEA-1.

#### Sixth Grade Students

Again, none of the students met the criteria of having an IQ equal or above 130 points in all three of the tests. Regarding the convergence between pairs of tests, the *Factor “g”* test and the TEA-1 converged, but only in a single case. The results of this student can be seen in **Table 4**. He showed a high level of creativity and his IQ was close to 130 when the Badyg-3 was administered.

In relation to students scoring 130 or above in each of the tests, results indicated that the TEA-1 was the test that identified the greatest number of students that met this criterion followed by the *Factor “g”* test. The Badyg-3 was the most restrictive test in this sense, as none of the students showed an IQ score equal to or higher than 130 in this test. **Table 4** presents the results corresponding to each group. In this case, 50% of the children identified as high-ability students in the different tests displayed a high level of creativity. This pattern was different from that found in the group of third grade students, where only 2 out of 20 (10%) of the students identified as having high-abilities showed high levels in this variable. IQ values ranged from 130 to 139 in the case of the students identified by the *Factor “g”* test, whereas all the students identified by the TEA-1 showed an IQ of 135. The student identified by the Badyg-3 had an IQ of 136.

In summary, out of the total of 236 students, 31 students (20 from third grade and 11 from sixth grade) were identified as having an IQ equal to or greater than 130, considering the different tests separately. This corresponds to 13.13% of the sample. There were only three cases in which two tests produced converging results, which equates to only 1.27% of all students evaluated. No convergence of results was found among the three measures of intelligence.

## Discussion

This study has two main objectives: analysing the relationship between intellectual capacities of a group of third and sixth grade students from Northern Spain; and to analyze the discriminatory value and congruence between different tests of intelligence traditionally used in the identification of high-ability students. In general, results point to the need to use different tests in the identification process, as well as to include complementary measures (i.e., creativity) to create a multi-criterial system for the detection of students who fall into this category (Renzulli, 2012).

### Intellectual Capacities Results of the Students and Congruence among Measures

In general, results indicated that both third and sixth grade students showed an average intellectual ability (close to 100 in most of the cases). Regarding congruence among the different intelligence measures used, it is important to note that all the tests administered to third grade students showed positive and significant correlations to one another. A moderate to high association between the *Factor “g”* test and the tests of educational and intellectual aptitudes, more related to academic performance (TEA-1 and Badyg), was found. However, for sixth grade students, significant correlations were only found between the Badyg-3 and TEA-1 (both assess general intelligence through those abilities related to learning and academic performance – or crystallized intelligence). Thus, when the same tests were administered to older students, the correlation between crystallized intelligence measures increased, while the association between crystallized and fluid intelligence measures decreased, or even disappeared. These results are consistent with those reported by Pérez and González (2007), who noted that the subscales with a greater cultural basis (and containing more elements of the school curriculum) functioned differently according to age, and showed more congruence as children grow up (and presumably as their knowledge increases).

In addition, regarding the accuracy of the tests detecting high-ability students, it should be noted that the congruence among the various measures examined was disturbingly low. In this sense, none of the students met the criterion of showing an IQ equal to or above 130 in all of the three measures that were administered in a concurrently. On the other hand, considering the different tests separately, 13.13% of the total sample corresponds with students who were identified as having an IQ equal to or greater than 130, when theoretical percentage expectation would be around 2%. Differences in the estimations provided by the different tests for a same student were high. This may point to important constraints regarding the validity of the tests that are being currently being used.

It could thus be assumed that, at earlier stages of development, the different types of intelligence tests can converge, with respect to findings. However, this convergence tends to decrease with age, and congruence only stays present in cases in which those abilities have been facilitated and boosted by on-going learning. These findings have some implications for practice. Specifically, the lack of congruence among intelligence measures (such as that identified in this study) may lead to misdiagnosis, preventing some students from receiving adequate support for their exceptional needs. Likewise, it is appropriate to highlight the need to use different tests of fluid and crystallized intelligence in the identification of high-ability students, always taking into consideration the students’ cognitive developmental stages.

### Discriminatory Value of the Measures Identifying High-Ability Students and Intellectual Results Differences of the Students Detected

It is necessary to highlight that a reliable evaluation is the basis for an early detection and tailored intervention, and that currently one of the most important concerns regarding higher abilities is that these students often do not receive recognition, and thus appropriate intellectual stimulation, at least in Spain (Calero and García-Martín, 2014). This can lead to a lack of interest, frustration, and failure at school, as well as have a negative effect on the development of self-worth and social acceptance (Kroesbergen et al., 2016) or result in behavioral problems in some cases. On the other hand, a false positive may push students toward overly demanding and frustrating processes that may exceed the limits of their capacity. A total of 31 students in the current study presented an IQ equal or above 130 when the different tests were used separately, which corresponds to 13.13% of the sample. This infers a clear over-estimation of high-ability students, if the acknowledged distribution of IQ in the general population is to be taken into account. When convergence between any two tests was considered, only three students were identified as being high-ability children, which corresponds to only 1.27% of the total sample.

In terms of creativity, students in sixth grade showed higher scores in this variable in comparison to third grade students. This result suggests that creativity may increase as students progress through the different stages of schooling, and draws attention to the need for researchers to conduct more comprehensive studies on what type of teaching methods favor or hinder creativity in the classroom.

**FIGURE 1:**
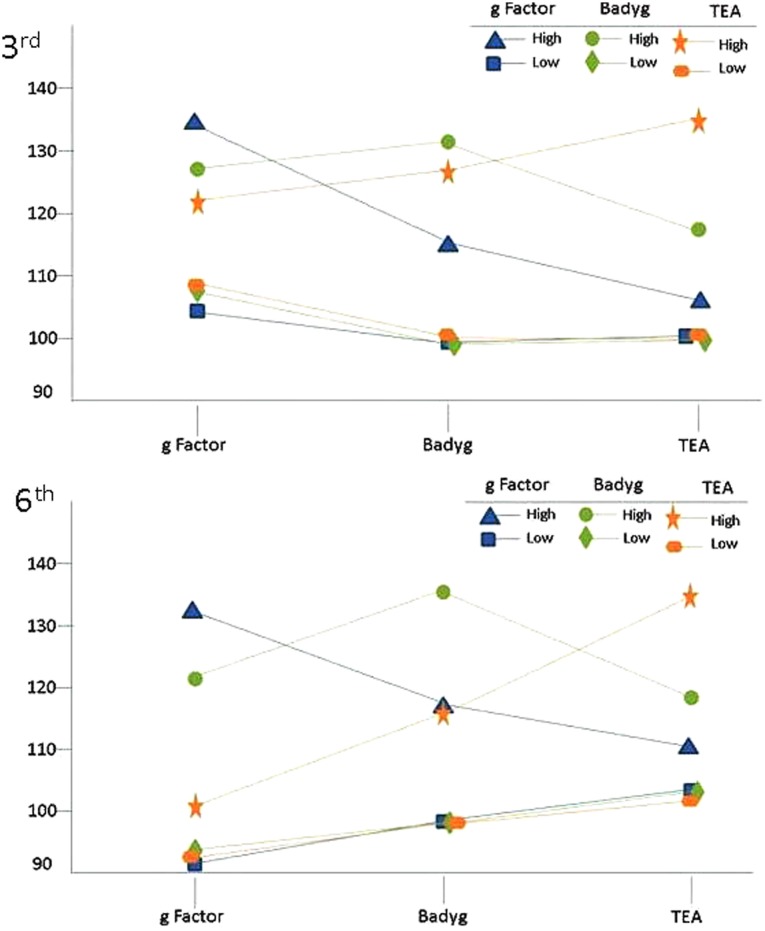
Results of the different measurements for groups with IQ higher and lower than 130 (depending on whether the IQ selection was made using *g Factor*, Badyg or TEA-1).

It is also worth noting some differences in the functioning of the tests according to grade level. Regarding third grade students, results suggest that the *Factor “g”* test may be less restrictive than the other tests when it comes to detecting potentially higher-ability students, whereas the TEA-1 may be the most liberal in this sense, identifying the greatest number of higher-ability students. However, the discriminating power of the tests in the case of sixth grade students was different. Specifically, the *Factor “g”* test and TEA-1 tests were the most and the least restrictive tests, respectively. Again it seems that the tests that measure fluid intelligence and those which measure crystallized intelligence operate differently at different developmental stages (see **Figure 1**).

With respect to the students’ intellectual variables, results indicated that a high IQ is not necessarily accompanied by high creativity, which has already been demonstrated in previous research (Kim, 2005; Marugán et al., 2010; Guignard et al., 2016). In the case of third grade students, 20 participants were identified as high-ability children by at least one of the tests. However, only two of them demonstrated high levels of creativity. Among the sixth grade students, only six of the 11 who were identified as high-ability students also displayed high levels of creativity. Studies carried out with large samples of Spanish students, such as that of Castejón et al. (2016), show how in classrooms, although gifted students are equally categorized, not all of them show the same cognitive-motivational profiles. In this way, there are students who exhibit higher scores on creativity and lower scores on general mental ability or self-regulation learning strategies (the group called by these authors as “creative gifted”) and there are student profiles that do not show special ability in this variable; for example, students called “gifted achievers,” who show high scores in self-regulation learning variables and academic achievement, and lower scores in creativity; or students called “cognitive gifted” who get high scores in general mental ability only.

In summary, and as Heller (2004), Ziegler and Stoeger (2010), and Wellisch and Brown (2012) have pointed out, the use of different tests of intellectual ability in the identification of high-ability students is necessary. Otherwise, this process may be biased. Furthermore, including additional measures not directly related to intellectual ability, such as creativity, would help establish a more detailed profile of the students and thereby assist in identifying their additional strengths and weaknesses. This is even more important in countries such as Spain, where most of the detection protocols available today, although multidisciplinary, still use a single measure of intellectual ability as a starting point to identify those students at a higher level of ability (Hernández and Gutiérrez, 2014). In this sense, it would be necessary to continue analysing the correspondence between different assessment tests, as well as between different measures of creativity, in order to better delimitate to what extent the tests provide a coherent and comprehensive profile of the students’ intellectual abilities.

Finally, some limitations should be acknowledged in relation to the present study. Firstly, the sample size was somewhat limited and also geographically localized, which may pose some constraints concerning generalization of the results. It would be necessary to expand the study sample to include a large number of gifted children and determine if the results obtained on the lack of congruence between the tests are maintained. In the current study, the percentage of students with scores above 130 IQ points appears biased toward the distribution or congruence between the measures. Secondly, future studies may consider the benefits of including additional variables in research of this kind, such as motivation, personality, learning styles, socio-cultural conditions, and/or students’ affective-emotional states. These additions to the methodology utilized in the present study would undoubtedly enhance the results of any future investigations of the multidimensional construct widely known as “higher ability” (Reis and Renzulli, 2010; Sternberg, 2010; Hernández and Gutiérrez, 2014). Finally, although through different tests, the same construct (IQ) has been evaluated. Thus, the possibility of an average regression effect or profiles with cluster latent analysis, which is common when evaluating students in a short period of time, has to be considered. It would be interesting to extend the time between evaluations in order to control for this effect in future research.

## Ethics Statement

This study was carried out in accordance with the recommendations of University of Oviedo with written informed consent from the parents of all participants. All parents gave written informed consent in accordance with the Declaration of Helsinki. The protocol was approved by the University of Oviedo.

## Author Contributions

EF, TG, and CR have participated in the design, analysis and drafting of the paper. OA-G, and AV have participated in the application of the measures and drafting of the paper.

## Conflict of Interest Statement

The authors declare that the research was conducted in the absence of any commercial or financial relationships that could be construed as a potential conflict of interest.
